# Urinary vanin-1 as a novel biomarker for survival in peripheral artery disease

**DOI:** 10.1177/1358863X241240428

**Published:** 2024-04-12

**Authors:** Bernhard Zierfuss, Anna Karlinger, Marija Bojic, Renate Koppensteiner, Gerit-Holger Schernthaner, Clemens Höbaus

**Affiliations:** 1 Medicine II, Division of Angiology, Medical University of Vienna, Vienna, Austria; 2 1st Medical Department, Hanusch Hospital, Vienna, Austria; 3 Medicine III, Division of Nephrology and Dialysis, Medical University of Vienna, Vienna, Austria

**Keywords:** chronic kidney disease, fibrosis, inflammation, mortality, peripheral artery disease (PAD), urinary vanin-1, biomarkers

## Abstract

**Background:**

Chronic kidney disease is associated with increased rates of incidence, morbidity, and mortality in lower-extremity peripheral artery disease (PAD). No specific marker for a functional risk assessment of kidney disease in PAD is known, especially at the early stages. Thus, we speculated that urinary vanin-1 (uVNN1), a marker of oxidative stress even in early kidney injury, could further stratify outcome assessment in patients with PAD.

**Methods:**

Patients with stable PAD (*n* = 304) of the Vienna medical cohort were followed up for up to 10 years and the outcome was assessed by central death database queries. uVNN1 was measured by enzyme-linked immunosorbent assay (ELISA) at study inclusion and normalized to urinary creatinine (uVNN1/Cr). During the observation time (9.3, 7.0–9.8 years), 104 patients died, 54.8% of which were due to cardiovascular causes.

**Results:**

uVNN1/Cr was associated with a urine albumin–creatinine ratio (UACR) (*R* = 0.166, *p* = 0.004) but not with an estimated glomerular filtration rate (*R* = 0.102, *p* = 0.077). Levels of uVNN1/Cr did not differ between asymptomatic and symptomatic PAD (*p* = 0.406). Kaplan–Meier curves showed a clear-cut association with higher all-cause (log-rank *p* = 0.034) and cardiovascular mortality (log-rank *p* = 0.032) with higher uVNN1/Cr levels. Similarly, significant associations for all-cause (hazard ratio [HR] 1.34, 95% CI [1.08–1.67], *p* = 0.009) and cardiovascular mortality (HR 1.45, 95% CI [1.06–1.99], *p* = 0.020) could be seen in multivariable Cox regression models.

**Conclusions:**

uVNN1/Cr showed an independent association with both all-cause and cardiovascular mortality in patients with PAD and was associated with early kidney disease. Thus, uVNN1 could be a useful marker for risk stratification of kidney disease in PAD.

## Background

Chronic kidney disease (CKD) is an important risk factor for the development of peripheral artery disease (PAD)^1,2^ and is an associated co-morbidity of PAD as well.^3^ Since the prevalence of PAD increases with age and the filtration capacity of the kidneys decreases with age, the majority of PAD patients exhibit at least a mildly reduced glomerular filtration rate.^4^ Furthermore, CKD and diabetes mellitus, which is highly co-prevalent with PAD, lead to a more distal (small artery disease) distribution pattern of atherosclerotic plaques^5^ and both, in addition, increase the prevalence of media sclerosis. In patients with PAD progression of CKD to end-stage renal disease (ESRD), expected survival is limited despite great advances in revascularization technique and medical therapy.^4,6^ Fortunately, only a minority of pa-tients with PAD progress to ESRD.^6^ Despite this relevant clinical problem, no adequate risk stratification tool for patients at excess risk for fatal events with the combination of CKD and PAD is in clinical use. Thus, a feasible measurable biomarker for this relevant problem is warranted.

Vascular noninflammatory molecule-1 (vanin-1; VNN1) expression is primarily found in the kidney, intestine, lung, and liver.^7^ VNN1 catalyzes the hydrolysis of pantetheine in pantothenic acid and cysteamine, thus recycling pantothenic acid for the biosynthesis of coenzyme A.^8^ VNN1 is regulated by peroxisome proliferator-activated receptor alpha (PPAR-α) in mice.^9^ Overexpression of VNN1 thus leads to oxidative stress response and fibrosis due to its involvement in these pathways.^10,11^

In addition, plasma triglycerides, low-density lipoprotein cholesterol (LDL-C), and inflammatory markers such as interleukin-1β increased in VNN1-treated mice and led to increased atherosclerotic plaques.^12^

Further animal models showed that urinary vanin-1 (uVNN1) is a possible biomarker for kidney injury^12^ and extrinsic ureteral obstruction.^13,14^ Likewise, uVNN1 is associated with markers parameters of CKD such as an estimated glomerular filtration rate (eGFR) category < 60 mL/min/1.72 m^2^ and a urine albumin–creatinine ratio (UACR) in humans.^15^ uVNN1 levels increase in patients with type 1 diabetes and macroalbuminuria.^16^ VNN1 has been proposed as a possible antioxidant response expressed in renal proximal tubular epithelial cells in mice models of acute tubular injury.^17^

Recently, uVNN1 levels were found to be associated with severity and incidence of coronary artery disease.^18^ These data show that uVNN1 is linked to both CKD and atherosclerosis. However, no data in patients with PAD are available, to our knowledge.

Thus, we speculated that uVNN1 could be used as a marker of increased mortality risk aggravated by kidney disease in PAD. uVNN1 was measured in patients with stable PAD to further elucidate the hypothesis of a possible association with long-term outcome.

## Methods

### Study cohort

uVNN1 levels were measured once in 304 patients with PAD from the Vascular Medicine Center (VMC) Vienna cohort. The VMC Vienna cohort consists of lower-extremity PAD patients (asymptomatic or claudication symptoms) without critical limb ischemia or planned revascularization at inclusion. PAD is used for the term LEAD – as proposed by the European Society of Cardiology (ESC) – to distinguish for lower-extremity artery disease.^19^ Patients with ulcerations, known cancer, or end-stage kidney disease were excluded, as previously reported.^20^ The study was approved by the ethics committee of the Medical University of Vienna and complied with the Declaration of Helsinki, including current revisions and Good Clinical Practice guidelines.^21,22^ The procedures followed were in accordance with institutional guidelines, and all subjects gave written informed consent before inclusion in the study.

### Patient evaluation

Patients were evaluated by standardized questionnaires including smoking status, body composition, and previous cardiovascular events. PAD was clinically assessed by vascular specialists and defined by an ankle–brachial index (ABI) below 0.9 or known previous peripheral revascularization. The Fontaine classification was used for the clinical assessment of self-reported pain-free walking distance. The ABI was assessed by trained technicians using noninvasi-ve Doppler sonographic measurements (ELCAT VL5000; Wolfratshausen, Germany). Mid-stream urine samples were collected in the first study year. Concurrently, blood samples were drawn for glycated hemoglobin A1c (HbA1c), cholesterol, liver, and renal function parameter measurement. After collection, serum and urine samples were aliquoted and stored at −80°C in a temperature-monitored freezer prior to analyses. Standard laboratory parameters were measured at the central laboratory of the General Hospital of Vienna. Urinary creatinine was measured with a Roche assay (Basel, Switzerland).

### Definition of comorbidities

Arterial hypertension was defined as the intake of any antihypertensive medication or systolic blood pressure of ⩾ 140 mmHg and/or diastolic blood pressure of ⩾ 90 mmHg in at least two measurements.^23^ Diabetes mellitus type 2 was defined as a fasting plasma glucose level over 7.0 mmol/L (126 mg/dL), a glucose level over 11.1 mmol/L (200 mg/dL) after a standardized oral glucose tolerance test,^24^ HbA1c over 6.5% (48 mmol/mol), or intake of antidiabetic medication. Former smoking was defined as the previous smoking of at least 100 cigarettes. Smoking one pack of cigarettes (20 pieces) per day was defined as one pack-year.

### Calculation of indexes

Body mass index (BMI) was calculated as body weight in kg divided by squared body height in meters (kg/m^2^). Spot UACR in > 30 mg/g were classified as micro-albuminuria and > 300 mg/g as macro-albuminuria. The eGFR was calculated by the Chronic Kidney Disease Epidemiology (CKD-EPI) equation.^25^ Patients were classified by kidney function using the eGFR (G1–G4) and UACR categories according to the 2012 Kidney Disease: Improving Global Outcomes (KDIGO) guidelines for the evaluation and management of CKD.^26^ Accordingly, patients were divided into four categories for the CKD progression risk, ranging from low risk (eGFR ⩾ 60 mL/min and UACR < 30 mg/g) to moderate risk (eGFR 45–59 mL/min, or eGFR ⩾ 60 mL/min and UACR 30–300 mg/g) to high risk (eGFR 30–44 mL/min and UACR < 30 mg/g, or eGFR 45–59 mL/min and UACR 30–300 mg/g, or eGFR ⩾ 60 mL/min and UACR > 300 mg/g) to very high risk (eGFR < 30 mL/min, or eGFR < 45 mL/min and UACR 30–300 mg/g, or eGFR < 60 mL/min and UACR > 300 mg/g), as stated in the current KDIGO guidelines.^26^ ABI was calculated according to the TASC criteria^27^ by dividing the higher ankle pressure by the higher brachial pressure. In the case of incompressible ankle arteries (ABI > 1.4), patients were classified as exhibiting media sclerosis.

### Outcome assessment

The date and cause of death were retrieved from the Austrian central death registry. *International Statistical Classification of Diseases, 10th revision* (ICD-10) codes were used from the central death registry and verified by hospital or autopsy reports, as available, to quantify cardiovascular mortality. Cardiovascular mortality was defined by the ICD-10 diseases of the circulatory system (I00–I99 codes). A total of 104 patients died during the 10-year observation period (54.8% were categorized as cardiovascular death).

### Measurement of vascular noninflammatory molecule-1 (VNN1)

Frozen urine samples were thawed at 4°C for VNN1 analysis; 304 urine samples were available for analysis. uVNN1 levels were measured from urinary samples from visits in the first year. Samples were prepared according to the assay protocol and measured by sandwich enzyme-linked immunosorbent assay (ELISA) with a sensitivity of 9.6 pmol/L (Biomedica, Vienna, Austria). The intra-assay and inter-assay coefficient of variation were 6.6% and 7.9%. The uVNN1 concentration was normalized to urine creatinine levels (uVNN1/Cr) according to Zamzam et al.^28^ to account for differences in hydration status.

### Statistics

Data are presented as mean ± SD or median (25, 75 percentile). Student’s unpaired *t*-test, as well as χ^2^ test, were used as appropriate. Differences between multiple groups were analyzed by analysis of variance (ANOVA) or Kruskal–Wallis test as appropriate. An alpha level of *p* < 0.05 (two-tailed) was considered statistically significant. In the case of nonnormal distribution, variables were log-transformed. Normal distribution was analyzed by the Kolmogorov–Smirnov test for parametric statistics if needed. VNN1 levels were log-transformed due to a left-skewed distribution and used for all further statistical evaluations, if not stated otherwise. Bivariate correlation was estimated using the Pearson correlation coefficient. Survival curves were calculated by the Kaplan–Meier method and compared using the log-rank test. Cox regression analysis was performed to estimate the effect size and to allow for multivariable adjustment. The multivariable adjustment was performed with traditional cardiovascular risk factors (age, sex, BMI, smoking status, eGFR, UACR, LDL-cholesterol, C-reactive protein, HbA1c). Two deceased patients were omitted in this analysis due to missing covariates. A simplified model in the multivariable Cox regression analysis of cardiovascular mortality was performed to account for the lower event rate and to avoid over-adjustment (age, sex, eGFR, and UACR). The effect size for uVNN1/Cr is given as hazard ratio (HR) per 1 SD and 95% CI. All statistical analyses were performed with the statistical software package IBM SPSS 28.0 (IBM Corp., Armonk, NY, USA). The figures were generated by GraphPad Prism (GraphPad Software Inc., La Jolla, CA, USA).

## Results

uVNN1 normalized to urinary creatinine levels (UVNN1/Cr) was evaluated in a cohort of 304 older patients with PAD (69 ± 11 years, 33.6% women). The PAD study cohort exhibited a high prevalence of arterial hypertension (91.8%), type 2 diabetes mellitus (44.1%), and active smoking (33.8%) as underlying risk factors for atherosclerosis development. Those risk factors were equally dist-ributed regarding uVNN1 tertiles, as shown in Table 1. Additionally, 25.9% exhibited intermediate and 21% high to very high risk for CKD progression. Most patients were on active treatment with statins (83.2%) and renin-angiotensin-aldosterone system blockade (RAAS; 73.7%). Urinary VNN1/Cr levels were not influenced by the patient’s age (*R* = 0.021, *p* = 0.711) or body mass index (*R* = −0.085, *p* = 0.140). In addition, uVNN1/Cr levels were not linked to metabolic parameters such as HbA1c (*R* = 0.108, *p* = 0.059), LDL-cholesterol (*R* = 0.067, *p* = 0.245), eGFR (*R* = 0.102, *p* = 0.077), or C-reactive protein (*R* = −0.011, *p* = 0.844). Urinary VNN1/Cr levels were higher in women (*p* < 0.017). Details are depicted in Table 1.

**Table 1.** Baseline characteristics according to uVNN1/Cr.

	Overall cohort	1^st^ tertile	2^nd^ tertile	3^rd^ tertile	*p*-value
*N*	304	101	102	101	
uVNN1/Cr, pg/L/mg/dL	2.79 (1.90–4.24)	1.50 (1.05–1.90)	2.79 (2.57–3.19)	5.10 (4.24–6.08)	< 0.001
Age, years	69 ± 11	68 ± 11	70 ± 10	70 ± 10	0.329
Women	102 (33.6)	25 (24.8)	33 (32.4)	44 (43.6)	0.017
Body mass index, kg/m^2^	27.4 ± 4.1	27.5 ± 3.9	27.8 ± 3.9	26.9 ± 4.4	0.283
HbA1c, mmol/mol	42 (38, 49)	42 (38, 46)	42 (38, 46)	42 (39, 53)	0.319
LDL-C, mmol/L	2.56 (2.07, 3.26)	2.53 (2.15, 3.26)	2.49 (2.07, 3.32)	2.64 (1.99, 3.23)	0.895
Statin usage	253 (83.2)	79 (78.2)	93 (91.2)	81 (80.2)	0.029
C-reactive protein, mg/L	3.0 (1.5, 5.7)	3.1 (1.6, 5.6)	3.1 (1.6, 6.9)	2.7 (1.4, 5.6)	0.567
eGFR, mL/min/1.73 m^2^	68 ± 19	67 ± 20	68 ± 17	69 ± 19	0.801
UACR, mg/dL	75 ± 233	34 ± 111	59 ± 251	133 ± 290	0.007
eGFR < 60	101 (33.1)	34 (33.7)	33 (32.4)	34 (33.7)	0.974
Hypertension	279 (91.8)	89 (88.1)	97 (95.1)	93 (92.1)	0.193
Diabetes mellitus Type 2	134 (44.1)	44 (44.4)	40 (39.2)	50 (50.0)	0.305
RAAS blockade	224 (73.7)	73 (72.3)	72 (70.6)	79 (78.2)	0.432
Smoking – active	103 (33.8)	40 (40.8)	36 (35.6)	27 (26.7.2)	0.106
Coronary artery disease	98 (32.1)	31 (30.7)	34 (33.3)	33 (32.7)	0.916
Carotid artery disease	124 (40.7)	38 (37.6)	43 (42.2)	43 (42.6)	0.729

Data presented as mean ± SD or median (25, 75 percentile) or *n* (%).

Differences were analyzed by ANOVA, Kruskal–Wallis, and chi-squared test between vanin-1 tertiles as appropriate. An alpha-level of *p* < 0.05 (two-tailed) was considered statistically significant.

eGFR, estimated glomerular filtration rate according to the Chronic Kidney Disease Epidemiology (CKD-EPI) equation; HbA1c, glycated hemoglobin A1c; LDL-C, low-density lipoprotein cholesterol; RAAS, renin angiotensin aldosterone system; UACR, urine albumin-to-creatine ratio; uVNN1/Cr, urinary vanin-1 normalized to urinary creatinine levels.

### uVNN1 and peripheral atherosclerosis

Clinically asymptomatic patients with PAD and those who reported claudication symptoms showed similar uVNN1/Cr levels (*p* = 0.406). PAD severity reflected by patients’ ABI was not associated with uVNN1/Cr levels (*R* = −0.030, *p* = 0.663, *n* = 213). Furthermore, the presence of media sclerosis (*n* = 81) did not result in increased uVNN1/Cr levels (0.42 ± 28 vs 0.48 ± 0.27, *p* = 0.100). Atherosclerosis extending to other vascular regions such as additional coronary or carotid artery disease did not alter uVNN1/Cr levels (*p* = 0.763).

### uVNN1 and renal function parameters

**Figure 1.**
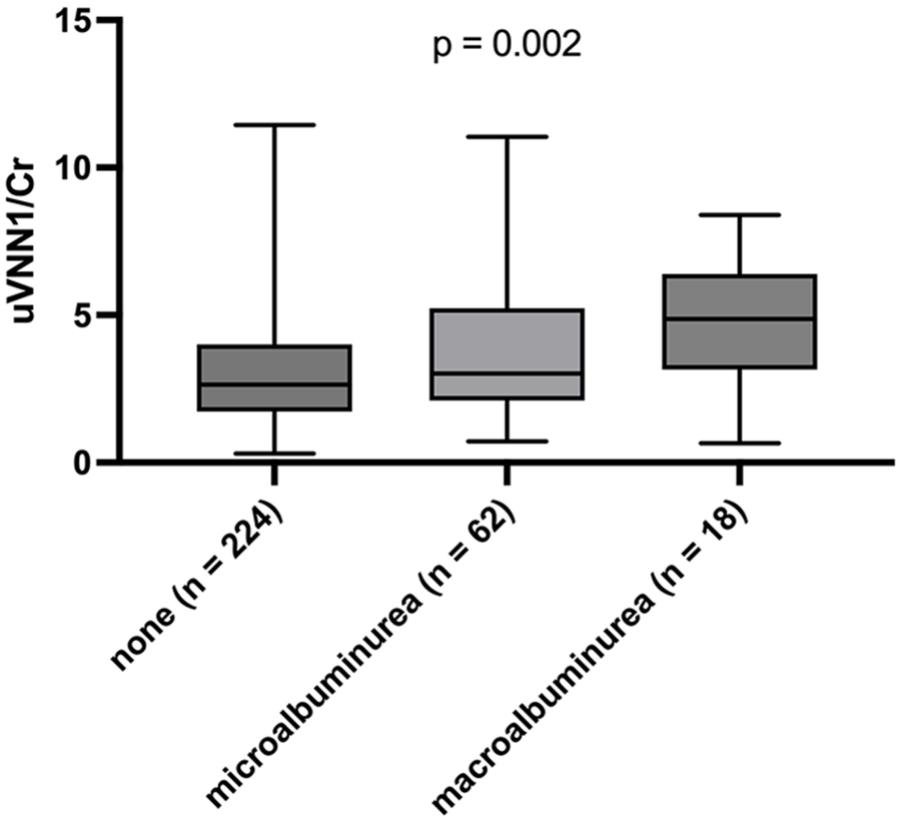
Urinary vanin-1 and albuminuria categories boxplot of uVNN1/Cr levels and albuminuria according to no albuminuria, micro-albuminuria (> 30 mg/g), and macro-albuminuria (> 300 mg/g). uVNN1/Cr, urinary vanin-1 normalized to urinary creatinine levels.

**Figure 2.**
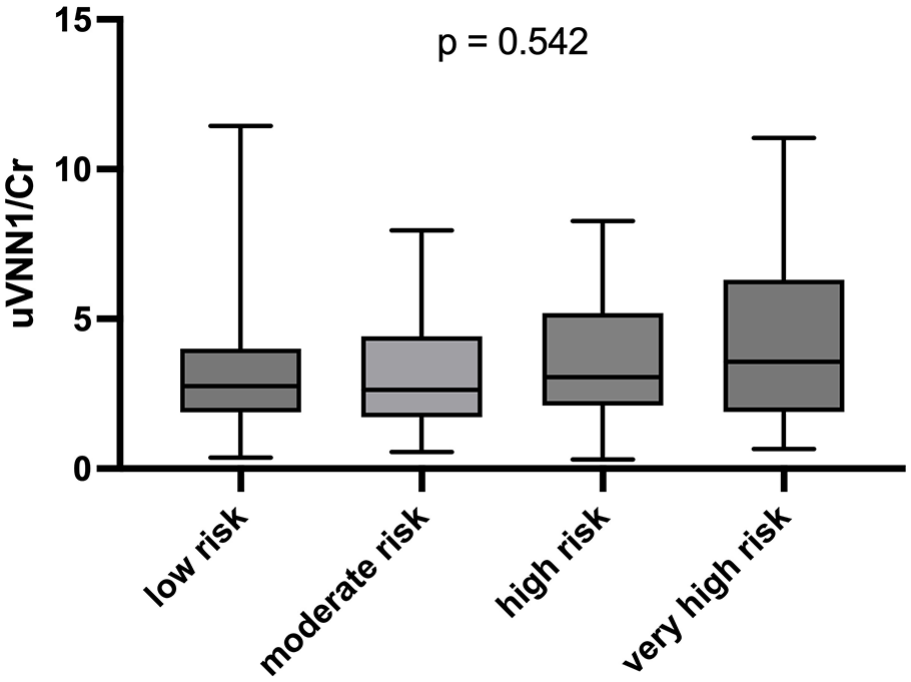
Urinary vanin-1 and risk of chronic kidney disease progression boxplot of uVNN1/Cr levels and KDIGO risk for chronic kidney disease progression category. KDIGO, 2012 Kidney Disease: Improving Global Outcomes; uVNN1/Cr, urinary vanin-1 normalized to urinary creatinine levels.

**Figure 3.**
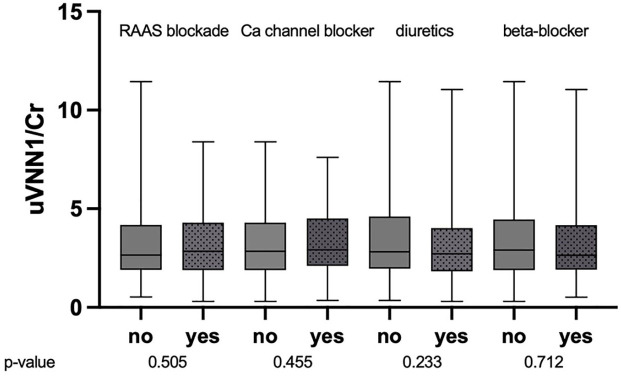
Urinary vanin-1 and antihypertensive agents boxplot of uVNN1/Cr levels and the intake of antihypertensive medication. RAAS, renin-angiotensin-aldosterone system blockade; uVNN1/Cr, urinary vanin-1 normalized to urinary creatinine levels.

uVNN1/Cr levels were significantly associated with patients’ UACR (*R* = 0.166, *p* = 0.004), but not eGFR (*R* = 0.102, *p* = 0.077). Figure 1 shows the association of uVNN1/Cr levels and the presence of micro- or macro-albuminuria (*p* = 0.002). The combination of both surrogate biomarkers for the estimation of CKD progression (eGFR and UACR) was not associated with uVNN1/Cr levels, as shown in Figure 2 (*p* = 0.542). In a cohort with overt hypertension, the use of RAAS blockade (*p* = 0.505) or any other antihypertensive agent did not change uVNN1/Cr levels, as shown in Figure 3.

### Urinary vanin-1 (uVNN1) and peripheral atherosclerosis

**Figure 4.**
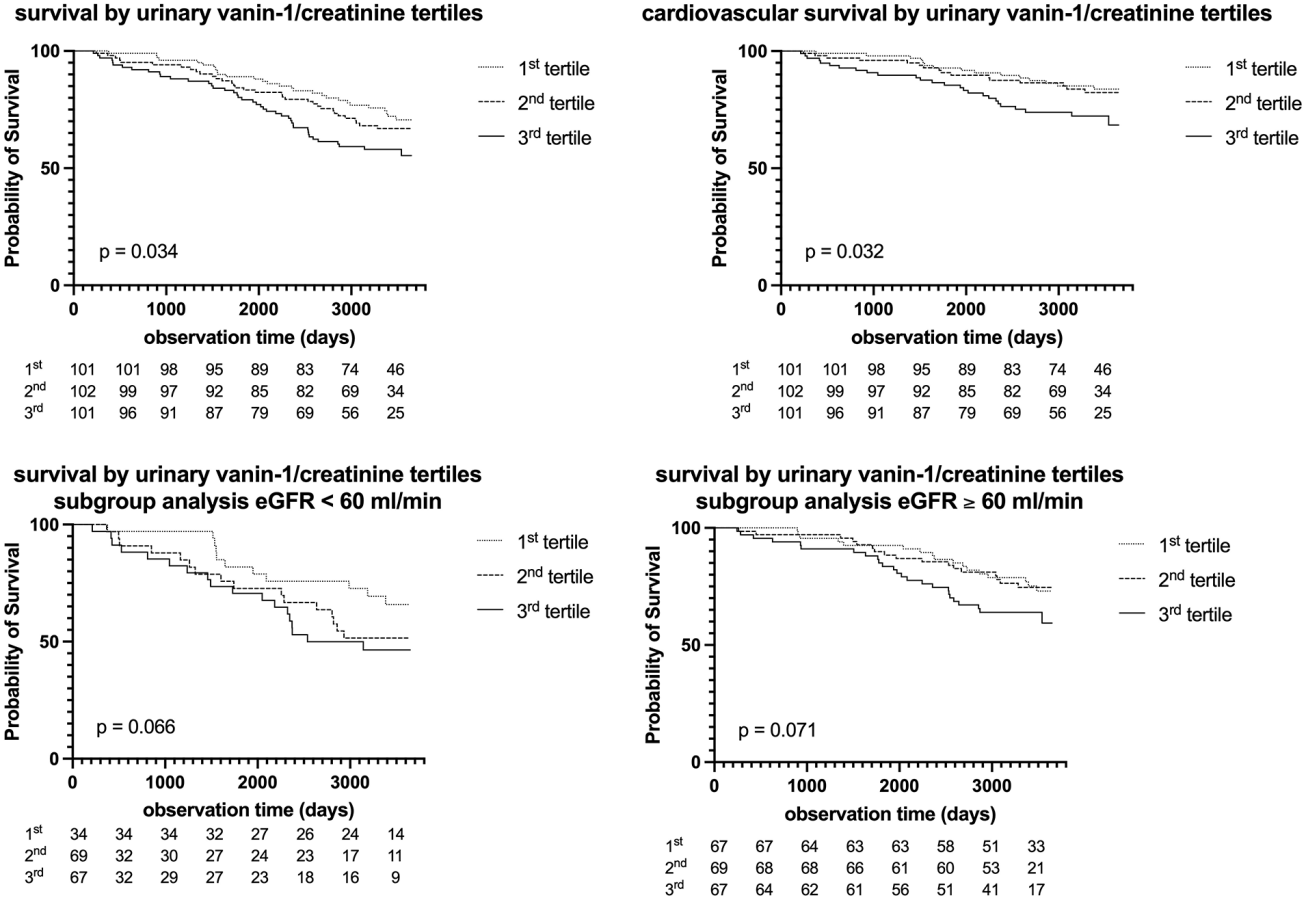
Urinary vanin-1 and long-term mortality Kaplan–Maier curve for the prediction of all-cause survival, cardiovascular mortality as well as subgroup analyses for eGFR < 60 and eGFR > 60 according to uVNN1/Cr over 10 years. Tertiles were compared using the log-rank test (*p*-value). eGFR, estimated glomerular filtration rate according to the Chronic Kidney Disease Epidemiology (CKD-EPI) equation; uVNN1/Cr, urinary vanin-1 normalized to urinary creatinine levels.

uVNN1/Cr levels were significantly associated with long-term all-cause mortality over 9.3 (7.0–9.8 years) in PAD patients (HR 1.33, [1.08–1.67], *p* = 0.009) as shown in Table 2. This link remained significant after multivariable adjustment for patient age, sex, BMI, and smoking status, as well as metabolic parameters including eGFR, UACR, LDL-cholesterol, C-reactive protein, and HbA1c (HR 1.29, 95% CI [1.03–1.63], *p* = 0.028). Furthermore, uVNN1/Cr levels were significantly associated with future cardiovascular mortality in PAD patients in univariate (HR 1.43, 95% CI [1.07–1.93], *p* = 0.017) and a multivariable-adjusted model for patients’ age, sex, eGFR, and UACR (HR 1.45, 95% CI [1.06–1.99], *p* = 0.020). Figure 4 depicts the Kaplan–Meier curves for all-cause (*p* = 0.034) and cardiovascular survival (*p* = 0.032) according to uVNN1 tertiles.

**Table 2.** Multivariable model of all-cause mortality and cardiovascular by uVNN1/Cr.

	All-cause mortality		
	Hazard ratio (CI)	Events	*p*-value
Unadjusted model	1.33 (1.08–1.67)	102	0.009
Multivariable model + patient age, sex, BMI, smoking, eGFR, UACR, LDL-C, CRP, HbA1c	1.29 (1.03–1.63)		0.028
	Cardiovascular mortality		
	Hazard ratio (CI)	Events	*p*-value
Unadjusted model	1.43 (1.07–1.93)	57	0.017
Multivariable model + patient age, sex, eGFR, UACR	1.45 (1.06–1.99)		0.020

Cox regression analyses for all-cause and cardiovascular mortality by uVNN1.

CRP, C-reactive protein; eGFR, estimated glomerular filtration rate according to the Chronic Kidney Disease Epidemiology (CKD-EPI) equation; HbA1c, glycated hemoglobin A1c; LDL-C, low-density lipoprotein cholesterol; smoking, smoking status; UACR, urine albumin–creatinine ratio; uVNN1/Cr, urinary vanin-1 normalized to urinary creatinine levels.

## Discussion

This study evaluates a possible relationship between uVNN1/Cr, as a marker of kidney disease, and PAD severity, as evaluated by ABI and Fontaine stage, and outcome. uVNN1/Cr was significantly associated with long-term outcome in a standard-sized cohort of patients with stable PAD. Multivariable Cox regression models withstood adjustment for traditional cardiovascular risk factors and standard markers of renal disease – UACR and eGFR. Levels of uVNN1/Cr were significantly higher in patients with both micro- and macroalbuminuria in contrast to patients without albuminuria. However, no association was seen with the risk of kidney progression (as assessed by the combination of eGFR and albuminuria) as well as eGFR per se. Restrictively, only patients with mild to moderate eGFR reduction were evaluated in this study.

In vitro and animal model studies could explain the findings of this study. With the hydrolytic activity of pantetheine into pantothenic acid and cysteamine, VNN1 exerts a major step in glutathione synthesis.^29^ Via this enzymatic activity, vanin-1 has been linked to oxidative stress response and fibrosis^10,11^ in several tissues but primarily in the kidney. VNN1 knockout mice showed reduced oxidative stress and thus reduced inflammation in the vasculature.^30^ In line with these findings are results from kidney injury mouse models. These models showed that overexpression of VNN1 was associated with poor renal repair and knockout models of VNN1 resulted in less kidney fibrosis after ischemia/reperfusion.^31^ Owing to the primary localization in the proximal tubular epithelial cells,^12,32^ VNN1 is postulated to be an early marker of damage in kidney disease in mice.^14^

A large proportion of patients with PAD suffer from co-prevalent CKD, at least at mild stages.^2^ CKD, even at stage 2, is associated with an increased incidence of PAD.^2^ A large study of about 30,000 patients found that patients with PAD and co-prevalent CKD are at a 74% higher risk of any major adverse cardiovascular events (MACE) after peripheral revascularization.^33^ However, eGFR, UACR, or a combination of both are only functional parameters to describe the level of kidney injury/disease, and no specific pathobiological pathway can be assessed this way. uVNN1/Cr represents a state of higher oxidative stress, possibly linking epithelial inflammation of the kidney to the development of CKD and thus, as we hypothesized, to accelerated atherosclerotic disease progression. The findings of the presented study are in line with this hypothesis. Since UACR and not eGFR was associated with uVNN1/Cr in our cohort, and uVNN1/Cr increases even at early stages of kidney disease/injury, even early changes of oxidative stress homeostasis dysregulation due to kidney disease could increase PAD progression. These findings on outcome of manifest atherosclerotic disease would be in line with the mentioned results of early kidney injury both in animal models^12^ and in humans.^15,34^ Furthermore, the effects seen by uVNN1/Cr secretion were not changed with different types of RAAS blockade or antihypertensive treatment, especially diuretics. This could indicate that uVNN1/Cr is independent of current pharmacologic intervention at both the glomerular and the tubular level. Furthermore, no previous study in both CKD and atherosclerotic cohorts could demonstrate an association with mortality. Another advantage of uVNN1/Cr can be seen in the feasible measurement method, which can be made combined with an UACR spot urine analysis with only an additional ELISA measurement method. UVNN1/Cr could thus represent a potential biomarker for excess mortality risk in patients with PAD and CKD. However, longitudinal data on intraindividual changes and relevance for outcome prediction are needed to further test for this hypothesis.

### Study limitations

Several limitations of this study must be considered. First, UACR measurement was only performed once. Second, only fatal outcome events were assessed. Third, no longitudinal data can be presented. However, several strengths must be considered as well. The size of cohorts for patients with PAD is representable. A long-term follow-up of up to 10 years was performed for outcome assessment and patients were managed with stringent guideline-directed therapy for traditional cardiovascular risk factors.

## Conclusion

In conclusion, uVNN1/Cr shows an independent association of both all-cause and cardiovascular mortality in patients with PAD. uVNN1/Cr could be a feasible and easily accessible marker for risk stratification of early kidney disease patients with PAD, which could further help to select patients with PAD at excess risk for fatal events.
